# Microbiological assessment of sachet water “pure water” from five regions in Ghana

**DOI:** 10.12688/aasopenres.12837.2

**Published:** 2019-01-24

**Authors:** Lydia Mosi, Samuel Mawuli Adadey, Sandra Akoley Sowah, Charles Yeboah

**Affiliations:** 1 West African Centre for Cell Biology of Infectious Pathogens, Department of Biochemistry, Cell and Molecular Biology, College of Basic and Applied Sciences, University of Ghana, Accra, P.O. Box LG 54, Ghana; 2 Australian Institute for Bioengineering and Nanotechnology , University of Queensland, Brisbane , QLD, Australia; 3 Safe Water Network, Accra, Ghana

**Keywords:** microbiology, sachet water, coliforms, E. coli, most probable number (MPN)

## Abstract

**Background: **Sachet water, popularly known as “pure water” has become an invaluable entity in most Ghanaian households. Despite its importance, there is no extensive nationwide investigations on its wholesomeness for consumption. The aim of this study was to determine the microbiological quality of 41 brands of sachet water sampled in 16 districts across 5 regions in Ghana.

**Methods:** The samples were analyzed for the presence of total and fecal coliform ( *Escherichia coli*) using the Colilert*- 18 Test Kit.

**Results:** Majority of the samples (56.09%) were excellent, 4.87% satisfactory and 14.63% suspicious. Ten samples (24.4%) were unsatisfactory. For the degree of fecal contamination, (85.56%) were satisfactory, four (9.76%) were suspicious, and two others (4.88%) were unsatisfactory. The contaminations observed could be attributed to poor sanitary conditions (during and/or after production) and failure of some production facilities to adhere to standard manufacturing practices.

**Conclusion:** Our data suggest that microbiological quality sachet water from some sources have not yet attained levels that make it absolutely pure and wholesome for consumption in many areas.

## Abbreviations

Ghana Water Company Limited (GWCL)

Most Probable Number (MPN)

Hazard Analysis Critical Control Point (HACCP)

## Introduction

The occurrence of packaging water into sachets popularly referred to as “pure water” is one of the most lucrative business ventures in some West African countries including Ghana ^1^. This business has gained much popularity and acceptance among the Ghanaian populace particularly because in the past, drinking water was sold in cups and plastic bags hand-tied at one end; a practice which was faced with a lot of sanitary issues ^2^. Currently, the exact numbers of sachet water companies is unknown, as new ones spring up almost daily. There are more unregistered producers than registered ones, with the current estimate of registered producers reaching 3,000 ^2^.

“Pure water” contains 500ml of water in a clear plastic bag that is electrically heated and sealed at opposite ends. Water used for “pure water” is mostly obtained from ground water, springs and potable pipe-borne water. Prior to packaging, the water goes through a number of treatment processes, mainly filtration, in an attempt to make it cleaner and safer for consumption ^3^. Most households and families depended greatly on tap water from the Ghana Water Company Limited (GWCL) for drinking and household activities including cooking ^1^. However, with the frequent shortages associated with the supply of potable water across the country, and the questionable quality of the water supplied, many households and families in Ghana have resorted to using “pure water” mostly for drinking and cooking purposes ^4^.

According to WHO guidelines, water for drinking must not present any significant risk to the health of the consumer over a lifetime of consumption ^5^. Neither should the consumption of such water present different sensitivities that may arise between life stages. Invariably, safe drinking water should be colorless and tasteless, free from harmful chemicals as well as other suspended materials and most importantly should be devoid of disease-causing organisms ^6^. Among many other concerns, the possibility of drinking water being the source of disease-causing organisms and related illnesses has been a huge hurdle to overcome, especially in parts of developing countries where availability, accessibility and affordability of potable and safe drinking water continues to be a challenge ^2, 5^.

Although the introduction of sachet water was intended to provide affordable and readily available safe drinking water for Ghanaians, investigations on its quality and wholesomeness for consumption have revealed considerable gaps especially with regards to microbial quality. Ngmekpele and Hawkins in 2015 analyzed the microbial and physicochemical properties of sachet water sold in Obuasi in the Ashanti region and found total coliform levels exceeding the WHO and the Ghana Standards Authority’s accepted levels for drinking water. In addition, fecal coliform was also detected in one of the samples.

In a study to investigate the bacteriological quality of sachet water produced and sold in Teshie-Nungua, a suburb known for perennial water shortages, Addo *et al.* (2009) reported sachet water sampled with suspicious microbial contaminations based on the most probable number (MPN) values. Fecal coliforms were detected in several samples while some of the samples were also contaminated with *Escherichia coli*. Given the vast number of people that rely on sachet water for their drinking needs, it is imperative that its quality is of the highest standard to avert any future waterborne outbreaks related to its consumption.

The quality of packaged water assessed in Nigeria showed some levels of microbial contamination ^6^. The results of the study indicated that bottled water has lower microbial load than sachet water. *E. coli, Clostridium perfringens* spore and fecal *Streptococcus* were the most common isolated microbe from the packaged water. A similar study on the quality of potable water in Benin showed that some of the drinking water tested had microbiological pollution exceeding the approved levels, hence making the drinking water not wholesome for consumption ^7^. Although packaged water is an improved source of drinking water, it is not totally free from microbial contaminations; hence the need for enhanced monitoring strategies to ensure that packaged water is always safe for human consumption ^8^.

In this study we sought to examine the microbiological quality of sachet water sampled across Ghana, with primary focus on fecal contaminations. Although there have been several similar studies in the country ^3, 4, 9– 11^, our study has a wider geographic coverage (with samples from five regions out of the ten regions in Ghana) including mostly peri-urban and rural settings compared to previous studies where mostly urban settings were considered.

## Methods

### Study sites and design

**Figure 1.**
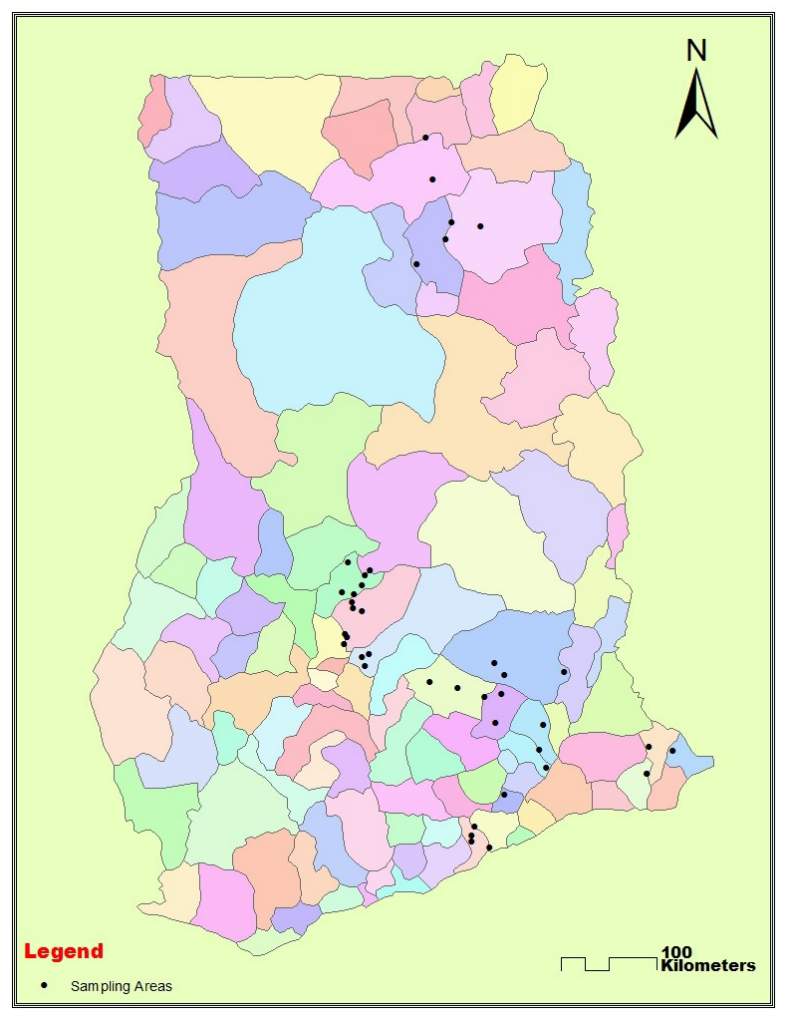
Map of Ghana showing the sites of sample collection. The dots represents the sample collection sites and illustrate the wide geographical coverage of the study.

Sachet water samples were collected from 41 selected communities within 16 districts in 5 out of the 10 regions in Ghana. The selection of the sample collection sites was based on a careful consideration of the sampling site of previous studies ^1, 3^ which were all based in the Greater Accra Region. The water samples were collected between May to June 2015. The communities were selected from the southern/coastal belt (comprising communities in Central and Volta Regions), the middle belt (comprising communities in the Ashanti and Eastern Regions) and the northern belt (comprising communities in the Northern Region) of Ghana. These communities gave a fair representation of rural, urban as well as peri-urban communities in Ghana thus giving the study a wider geographic coverage ( Figure 1). The selected communities comprised towns and villages where sachet water is sold by retail shops and hawkers ^12^. The study was designed to evaluate the microbial quality at the point of consumption of the most commonly sold sachet water.

### Sample collection

A total of 41 branded sachet water samples; one from each community (from different sachet water manufacturing companies), were randomly purchased from vendors on the streets. For each brand of sachet water, we tested 3 independent samples to correct for bias in sampling. The choice of purchase was based on the most commonly patronized sachet water by the inhabitants of a given community. The most commonly patronized sachet water was determined after interviewing a number of people in the community. The samples were stored on ice and transported (as described by *Johnson et al.*, ^7^) to central point laboratories within each region where the sample analyses were carried out.

### Sample inoculation and incubation

**Figure 2.**
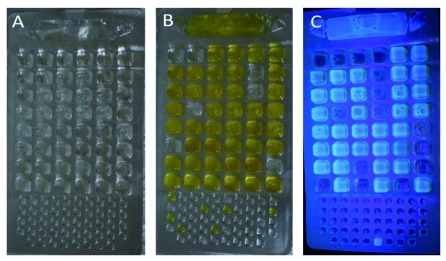
Total coliform and *Escherichia coli* enumeration: Quality control test for ( **A**) *Pseudomonas aeriginosa* (ATCC 10145), ( **B**) *Klebsiella pneumonia* (ATCC 31488) and ( **C**) *Escherichia coli* (ATCC 25922) to confirm negative result for both total coliform and fecal *E. coli,* positive results for total coliforms and positive results for fecal *E. coli* respectively. The positive wells for *E. coli* were observed under a 6-watt, 365-nm UV light in the dark.

We analyzed samples for the presence of total as well as fecal coliform ( *E. coli*) using the Colilert*-18 Test Kit (with catalog number WP2001-8 from IDEXX Laboratories, Inc., Westbrook, Maine, USA) following the manufacturer’s protocol. A set of quality controls were run for the lot of sachet water sampled within each region ( Figure 2). Briefly, ATCC strains of *Escherichia coli* (ATCC 25922), *Klebsiella pneumonia* (ATCC 31488) and *Pseudomonas aeriginosa* (ATCC 10145) were each transferred with sterile loops into three sterile containers coated with sodium thiosulfate and each filled with 100 ml of sterile water. A similar set up with deionized water and Colilert*-18 substrate only served as negative control. After 18 hours of incubation at 35°C, each Quanti-Tray was compared with a Quanti-Tray comparator for the presence or absence of total coliform. *E. coli* enumeration was performed by observing wells under a 6-watt, 365-nm UV light in the dark. The results were read following the interpretations in Table 1. Aseptic techniques were strictly followed to prevent laboratory based contaminations and this was confirmed by the negative controls.

**Table 1.** Quanti-Tray result interpretation for presence/absence of total coliform and *Escherichia coli*.

Appearance	Result
Yellow less than comparator	Negative for total coliform and *E. coli*
Yellow equal to or greater than comparator	Positive for total coliform
Yellow and fluorescence equal to or greater than	Positive for *E. coli*

The number of positive wells in each Quanti-Tray was counted and the corresponding Most Probable Number (MPN) was obtained from the MPN table ( https://www.idexx.com/pdf/en_us/water/qt97mpntable.pdf) provided by the manufacturer. With reference to the work done by Addo *et al*. in 2009, the WHO [4] and U.S. FDA ^13^ standards, the MPN values were used to categorize the samples. 

### Statistical analysis

Proportions of sachet water samples that were positive for *E. coli* and total coliform were compared across the five regions using Chi-square test with Yates correction for continuity using Microsoft excel (Microsoft Office 2013). The Marascuilo’s test of equality of several proportions was then used to compare pairs of proportions where the Chi-square test rejected the null hypothesis.

## Results

### Sachet water microbiological quality

**Figure 3.**
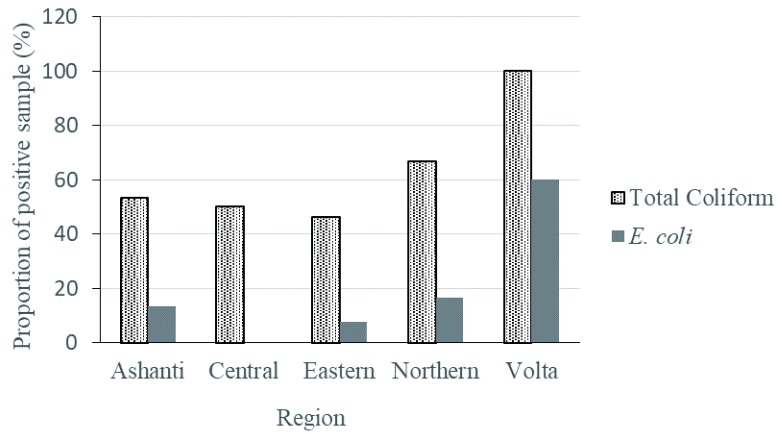
Microbial content of sachet water from five Regions in Ghana. Of the 41 sachet water sampled, 58.5% tested positive for the presence of total coliforms while 17.1% of the total coliform- positive samples also tested positive for fecal *Escherichia coli*.

One branded sachet water with the most patronage was collected in triplicate from each of the 41 communities. Twenty-four (58.5%) samples tested positive for the presence of total coliforms with 7 (17.1%) of the positive samples positive for fecal *E. coli.* No *E. coli* was detected in the Central Region samples. The highest MPN for total coliform and fecal *E. coli* was estimated at 1299.7 and 27.5 respectively with samples from the Volta region having more positive total coliforms and *E. coli* compared to other regions ( Table 2). However, results from Chi-square and Marascuilo tests showed that, ** the proportion of sampled sachet water that was positive for *E. coli* was significantly different across the five regions while the proportion that were positive for total coliform did not differ significantly across the five regions ( Figure 3 and Supplementary File 1; Supplementary Table 1 and Supplementary Table 2).

**Table 2.** Total coliform and *Escherichia coli* contamination of sachet water from 41 communities in Ghana.

Region	District	Community	*Total Coliforms*	Fecal *E. coli*
			MPN/100mL	MPN/100mL
Ashanti	SEKYERE WEST	WORASO	325.5	1
Ashanti	SEKYERE WEST	APAAH	2	<1
Ashanti	SEKYERE WEST	ADIDWAN	1	<1
Ashanti	SEKYERE WEST	BUNUSU	<1	<1
Ashanti	SEKYERE WEST	NINTING	2	<1
Ashanti	SEKYERE WEST	MPRIM	24.3	<1
Ashanti	SEKYERE EAST	NAAMA	<1	<1
Ashanti	EJURA SEKYEDUMASE	KOBRITI	<1	<1
Ashanti	EJURA SEKYEDUMASE	AFRAMSO	<1	<1
Ashanti	EJURA SEKYEDUMASE	BABASO	325.5	27.5
Ashanti	EJURA SEKYEDUMASE	MBANAA	<1	<1
Ashanti	SEKYERE EAST	OGUAA	1299.7	<1
Ashanti	EJURA SEKYEDUMASE	KASEI	<1	<1
Ashanti	EJURA SEKYEDUMASE	HIAWOANWU	<1	<1
Ashanti	SEKYERE EAST	ABOTANSO	155.3	<1
Central	AWUTU-EFFUTU-SENYA	OSIMPO 1	1.5	<1
Central	GOMOA	GOMOA LOME	<1	<1
Eastern	AKWAPIM SOUTH	FOTOBI	3.1	<1
Eastern	AKWAPIM SOUTH	OTU KWADJO	<1	<1
Eastern	YILO KROBO	TROM	<1	<1
Eastern	MANYA KROBO	AKOKOMA SISI	<1	<1
Eastern	MANYA KROBO	OBORPAH EAST	1	<1
Eastern	KWAHU SOUTH	NTESO	50.4	<1
Eastern	FANTEAKWA	ASIREBUSO	3.1	<1
Eastern	FANTEAKWA	ODUMASI	<1	<1
Eastern	FANTEAKWA	MPAEM	<1	<1
Eastern	KWAHU SOUTH	SUMINAKESE	6.3	<1
Eastern	KWAHU NORTH	KWAME DWAMENA	<1	<1
Eastern	KWAHU NORTH	FOSO (KWAWU FOSO)	920.8	<1
Eastern	KWAHU NORTH	KOKROBUTA/ ADAMUKOPE	<1	<1
Northern	SAVELUGU-NANTON	SAVELUGU TOWNSHIP	5.2	<1
Northern	WEST MAMPRUSI	LOAGRI NO.2	<1	<1
Northern	WEST MAMPRUSI	ARIGU	64	1
Northern	KARAGA	TONG	5.2	<1
Northern	KARAGA	KPATARIBORGU	<1	<1
Northern	KARAGA	TAMALEGU	4.1	<1
Volta	KETU	TADZEWU	5.2	<1
Volta	AKATSI	WUTE	14.5	<1
Volta	KETU	HEDZRANAWO	721.5	4.1
Volta	SOUTH TONGU	AGBAKOPE	9.7	3
Volta	SOUTH TONGU	AGBOGBLA	2	1

**Figure 4.**
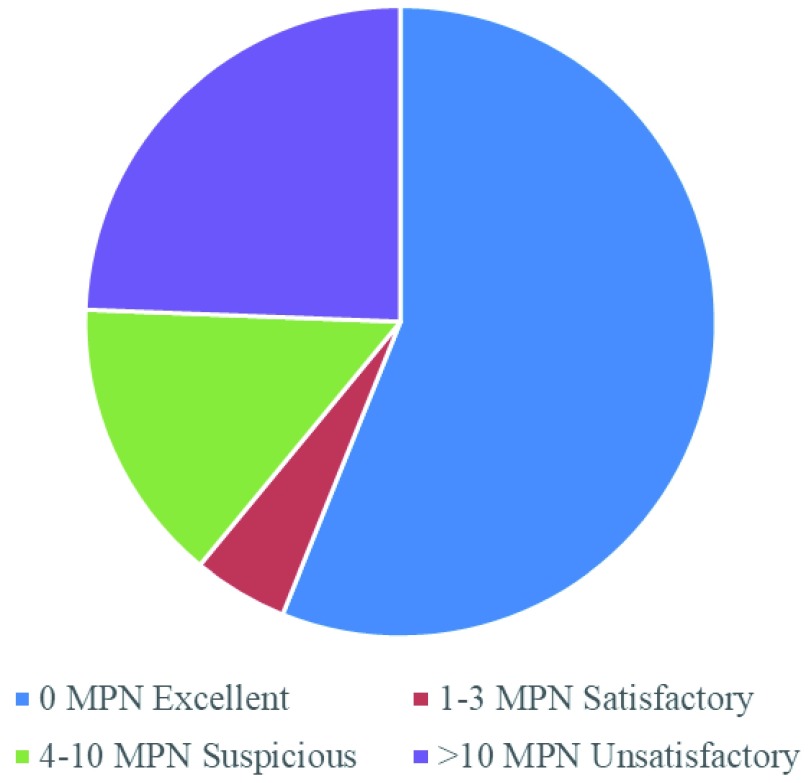
Grading of sampled water based on total coliforms. Classification of sampled water for total coliform contamination was based on the total coliform MPN according to Addo *et al*. (2009), WHO (2011) and U.S. FDA standard for water purity. Approximately fifty six percent (56.09%) were excellent, 4.87% and 14.63% were satisfactory and suspicious respectively. The remaining samples (24.41%) were unsatisfactory.

The results indicated that 11 out of the 41 (26.83%) sachet water brands sampled had coliforms exceeding the World Health Organization (WHO) and U.S. Food and Drugs Administration (U.S. FDA) approved limits of 9.2 MPN. The coliform MPN of the samples was used to grade the samples as Excellent (<2MPN/100), Satisfactory (2--3 MPN/100ml), Suspicious (4--10 MPN/100ml) and Unsatisfactory (>10MPN/100ml) ( Figure 4) ^3, 5^. The majority of the samples (56.09%) were excellent, with 4.87% and 14.63% as satisfactory and suspicious respectively. 10 samples (24.4%) however, were unsatisfactory ( Figure 4).

**Figure 5.**
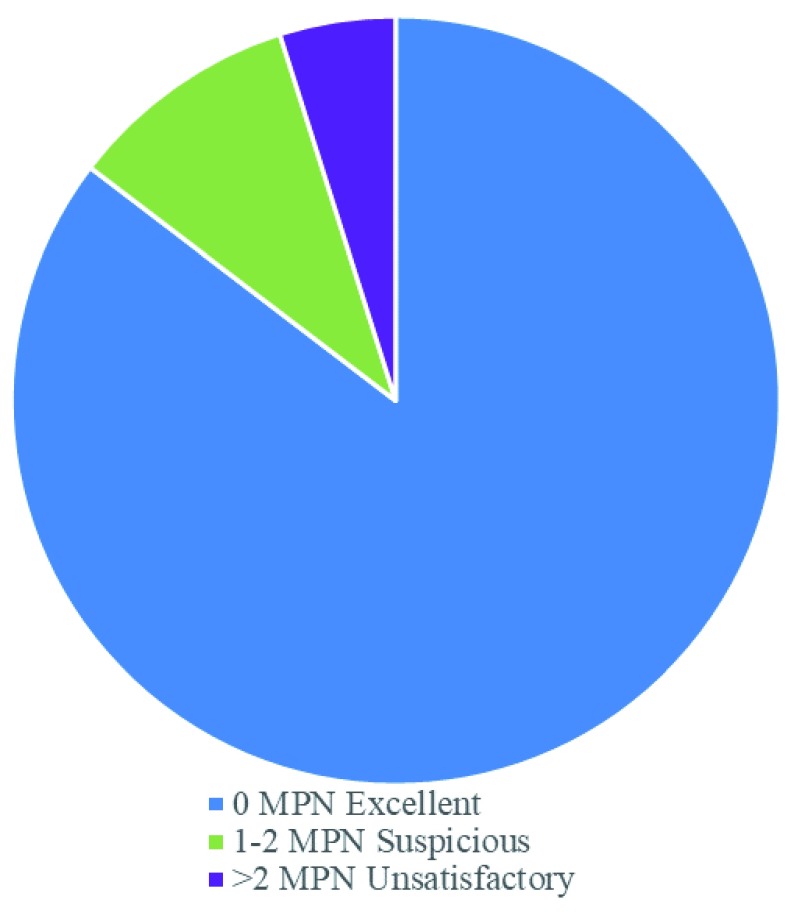
Grading of water samples based on total fecal *Escherichia coli.* Classification of sampled water for *Escherichia coli* contamination was based on the fecal *E. coli* MPN according to Addo *et al*. (2009), WHO (2011) and U.S. FDA standard for water purity. Majority of the samples (85.56%) were satisfactory, 9.76% were suspicious and 4.88% were unsatisfactory.

To determine the degree of fecal contamination, WHO and U.S. FDA standards were used to sort the samples based on the total fecal *E. coli.* The samples were graded Excellent (0 MPN/100ml), Suspicious (1--2 MPN/100ml) and Unsatisfactory (>2 MPN/100ml). The majority of the samples (85.56%) were satisfactory, however, 4 samples (9.76%) were suspicious and 2 others (4.88%) were unsatisfactory ( Figure 5).

## Discussion

The study of the microbiological quality of sachet water has been a topic of interest to researchers since mid-1990s ^14^. Nonetheless, publications on the microbial content of sachet water are still scarce, with only a few studies having a large sample size and are not representative of a nationwide study. Our study collected branded sachet water samples from 41 communities within 5 regions in Ghana to provide a better representation of the microbial water quality across the nation.

Based on the WHO ^5^ and U.S. FDA ^13^ standards, analytical samples should not have total coliform more than 9.2 MPN/100ml of water and must be free from fecal *E. coli* (thus 0 MPN/100ml). Our study revealed that 26.83% of our samples tested did not meet the above requirement as far as total coliform is concerned while 14.63% of the samples tested positive for fecal *E. coli*. Majority of the samples that had microbial contaminants were from the rural and peri-urban communities. These communities are often associated with poor sanitary conditions which could be a probable contributor to the above observations as well as failure of some production facilities to adhere to good sanitation practices. However, it is very important to further investigate the source of these contaminations.

Ideally, treated water should not have any coliform ^10^, however, several studies have indicated the presence of microbial contamination in sachet water from different parts of the country ^10, 11, 14, 15^. Although the sachet water samples collected for this study were presumptively treated by the manufacturer, 58.5% of the samples tested positive for total coliforms which is indicative of the risk associated with their consumption. The presence of fecal *E. coli* in these samples point to fecal contamination. The trend observed in the contamination across the regions suggests more of a generalized rather than a centralized contamination. These microbial contaminants could have been either introduced during the manufacturing or post-manufacturing processes. Should these contaminants be as a result of gaps in the manufacturing processes, then it raises a lot of concern about the efficiency of the treatment processes involved in making the water wholesome for consumption.

We recommend that further studies be carried to investigate the efficiency of the treatment processes. Ngmekpele & Hawkins (2015) attributed the failure of most sachet water companies to adhere to the Hazard Analysis Critical Control Point (HACCP) system as another cause of contamination. The HACCP seeks to help check and eliminate the various levels of contamination that may occur in the sachet water production processes. Therefore, it is very essential that the manufacturing of sachet water be closely monitored by the regulatory bodies in charge to ensure strict adherence to the standard manufacturing procedures. Addo *et al.* (2009) randomly collected 30 sachets of 10 different brands of “pure water” from Teshie and Nungua, in the Greater Accra Region of Ghana. From their analysis none of the brands sampled met the WHO standards for drinking water based on their microbial contamination. Also, other related studies ^10^ implicated both vendors and poor production practices as the source of the microbial contamination. To effectively identify the sources of contamination of sachet water, a progressive study should be done with different brands of sachet water following them from production to consumers.

The strengths of this study include (1) obtaining random samples from each of the five regions, (2) accounting for overestimation of statistical significance for small samples using Yates correction for continuity in the Chi-squared test, and (3) the use of fisher’s exact test where cell counts are less than five.

## Conclusion

The study identified microbial contamination; most alarming being contamination with *E. coli* in sachet water sampled in five regions across Ghana. As far as microbiological quality is concerned, sachet water has not yet attained levels that make it absolutely pure and wholesome for consumption.

## Regulatory bodies in Ghana

There are two main regulatory bodies in Ghana that are responsible for the regulation of companies that produce drinking water. Ghana Food and Drugs Authority ( https://fdaghana.gov.gh) which provides operational guidelines including codes of practice and good manufacturing practices for food, drug and other related manufacturing companies. The second regulatory body is Ghana Standards Authority ( https://www.gsa.gov.gh/) which has the vision of contributing towards growth of industry, protect consumers and facilitate trade through standardization, metrology and conformity assessment. These two regulatory ensure that drinking water producers meet all requirements for production before a permit is given. The regulatory bodies also have Quality Assessment departments that are responsible for quality check of licensed products including water. All the above efforts are towards the production of safe products for customer consumption.

## Data availability

Underlying data for this study is available from Open Science Framework: Dataset 1. Microbiological assessment of sachet water “pure water” from five regions in Ghana. http://doi.org/10.17605/OSF.IO/J968K ^16^ under a CC0 1.0 Universal license

## Supplementary material

**Supplementary File 1 – file containing the following Supplementary Tables:**

Supplementary Table 1: Chi-Square Test

Supplementary Table 2: Marascuilo test for *Escherichia coli* positive proportions
