# Supplementary material for: Microbiological assessment of sachet water “pure water” from five regions in Ghana
Source: AAS Open Res. 2019 Jan 24;1:12. Originally published 2018 Apr 18. [Version 2] doi: 10.12688/aasopenres.12837.2 (PMC7117957; doi:10.12688/aasopenres.12837.2)
Supplement: Supplementary file 1 [file aasopenres-1-14017-s0000.tgz › ae35306c-b738-45e7-ac6f-3ff440ddc98b.doc]

Supplementary Table 1: Chi-Square Test

|  | Total Coliform | E. coli |  |
| --- | --- | --- | --- |
| Critical Value | 9.487729037 | 9.487729 |  |
| Chi-Square Test Statistic | 5.279010281 | 17.01604 |  |
| p-Value | 0.259848264 | 0.001919 |  |
|  | Do not reject the null hypothesis | Reject the null hypothesis | |

The null and the alternative hypothesis used were as follows:

$$H_{o}: proportion of postives in Ashanti=proportion of postives in Central=proportion of postives in Eastern=proportion of postives in Northern=proportion of postives in Volta region$$

$$H_{a}:at least one of the proportions is different from the rest$$

Supplementary Table 2: Marascuilo test for *E. coli* positive proportions

| Proportions | Absolute Differences | Critical Range | interpretation |
| --- | --- | --- | --- |
| Ashanti - Eastern | 0.87 | 0.27 | Significant |
| Ashanti - Central | 0.13 | 0.27 | Not significant |
| Ashanti - Northern | 0.03 | 0.54 | Not significant |
| Ashanti - Volta | 0.47 | 0.73 | Not significant |
| Eastern - Central | 1.00 | 0.00 | Significant |
| Eastern - Northern | 0.83 | 0.47 | Significant |
| Eastern - Volta | 0.40 | 0.67 | Not significant |
| Central - Northern | 0.17 | 0.47 | Not significant |
| Central - Volta | 0.60 | 0.67 | Not significant |
| Northern - Volta | 0.43 | 0.82 | Not significant |
