# Supplementary material for: Microbiological assessment of sachet water “pure water” from five regions in Ghana
Source: AAS Open Res. 2019 Jan 24;1:12. Originally published 2018 Apr 18. [Version 2] doi: 10.12688/aasopenres.12837.2 (PMC7117957; doi:10.12688/aasopenres.12837.2)
Supplement: Peer review history

# Peer review: Reviewer response for version 2

**DOI:** 10.21956/aasopenres.14017.r26742

**Published:** 2019-02-04

**Authors:** Jonas A. Kengne-Ouafo, Bertrand Tatsinkou

**Affiliations:** 1 Department of Microbiology and Parasitology, University of Buea, Buea, Cameroon; 2 Department of Microbiology and Parasitology, University of Buea, Buea, Cameroon

The authors have addressed the questions and comments raised. I, therefore, recommend the manuscript for indexing.

We confirm that we have read this submission and believe that we have an appropriate level of expertise to confirm that it is of an acceptable scientific standard.

# Peer review: Reviewer response for version 2

**DOI:** 10.21956/aasopenres.14017.r26741

**Published:** 2019-01-31

**Authors:** Olusegun A. Olaoye

**Affiliations:** 1 Department of Food Science and Technology, Michael Okpara University of Agriculture, Umudike, Nigeria

Authors have made significant improvement in the revised version of the manuscript based on reviewer's comments. I recommend acceptance of the manuscript for indexing.

I confirm that I have read this submission and believe that I have an appropriate level of expertise to confirm that it is of an acceptable scientific standard.

# Peer review: Reviewer response for version 1

**DOI:** 10.21956/aasopenres.13903.r26349

**Published:** 2018-05-03

**Authors:** Jonas A. Kengne-Ouafo, Bertrand Tatsinkou

**Affiliations:** 1 Department of Microbiology and Parasitology, University of Buea, Buea, Cameroon; 2 Department of Microbiology and Parasitology, University of Buea, Buea, Cameroon

Findings presented in this paper are of great public health importance.  Mosi *et al *assessed the microbial content of different brands of sachet water produced, sold and consumed by the majority of the populations in different regions of Ghana with emphasis laid on coliforms and fecal E. coli. They showed that some sachet water brands in Ghana as a whole (since they sampled from 5 geographical distinct regions) are still contaminated by coliforms and fecal E. coli despite awareness-raising from previous reports. However, it would be good to specify the date of water sample collection in the study design to attest that what are referred to as previous reports are really previous reports in term of sample collection period. I will recommend this paper for indexing provided the authors address the comments raised below.

**Introduction**

 The authors present sachet water production and consumption at the beginning of the introduction as something specific to Ghana whereas it is common practice in many countries in Africa (West Africa mainly) (Nigeria, Cameroon). It would be good to move from general to specific.

**Methods**

 Figure 1 and Table 1 are used in the method section to back up the wider geographic coverage of this study. But there is no link between what is found in table 1 and geographical coverage. The authors should clarify that. Was the map drawn by the authors or it is just an adaptation from another map? If that is the case, then the authors should specify that.

**Study site and design**

 Sachet water samples were collected from 41 selected communities. How many samples were collected per community? The reader may have the impression that only one sample was collected per community. If that is the case, why? If many samples were collected then, your sample size should be 41 times the number of sample per brand. The authors should have collected more samples per community and even evaluate the storage conditions in each store, which information would have helped them discuss better their results. Did the authors collect each sachet water brands from the source (packaging company) to rule out the fact that water leaves the company already contaminated? If not, they should put that as a point in the discussion.

 Giving the brand’s names as Obiri‐Danso *et al* (2003) ^1^ did in their paper “The microbiological quality of drinking water sold on the streets in Kumasi, Ghana” could be of great importance to the consumers in making the choice of which brands to take. Maybe ethically this is not correct but the authors should think about it.

 “These  communities  gave  a  fair  representation of  rural,  urban  as  well  as  peri-urban  communities  in  Ghana  thus giving  the  study  a  wider  geographic  coverage “  the authors mentioned this in their study design, the reader would expect them to present the results and discuss them with respect to level of urbanisation which is not the case in the current version of the paper.

**Sample collection**

 -There is a word missing in the following sentence “The most commonly patronized sachet

 water  was  determined  after  interviewing  a  number  of  people the  community [...] to central point laboratories within each region where analysis were carried out." There seems to be a grammatical error in this sentence “where analysis was carried out or where analyses were”…

 Again, in the methods, the authors did not describe clearly the measures taken to avoid contamination during sample transport and manipulation in the lab which could have contributed to the number of micro-organisms found.

**Results**

 ……E.  coli was estimated  at  1299.7  and  27.5  respectively  with  samples  from the Volta region having more positive total coliforms and E. coli compared  to  other  regions  (Table  1)

 There is no link between the sentence and table1. The authors should make sure tables and figures are properly reference in the text.

 The title of figure 3 :”Regional analysis of the samples” does not really describe the figure. The reader may think from the title that the figure summarizes the methods used to analyse the sample in different regions and not the analysis outcomes which the authors are referring to.

**Discussion**

 The authors could consider adding a small paragraph on the impact of used sachets on the environment, e.g. they constitute breeding sites for mosquitoes such as anopheles which transmit other diseases.

We confirm that we have read this submission and believe that we have an appropriate level of expertise to confirm that it is of an acceptable scientific standard, however we have significant reservations, as outlined above.

## References

[1] : The effects of UVB and temperature on the survival of natural populations and pure cultures of Campylobacter jejuni, Camp. coli, Camp. lari and urease-positive thermophilic campylobacters (UPTC) in surface waters. *Journal of Applied Microbiology*.2001;90(2) : 10.1046/j.1365-2672.2001.01239.x 256-267 10.1046/j.1365-2672.2001.01239.x 11168729

# Response

**Published:** 2019-01-07

**Authors:** Samuel Mawuli Adadey

**Affiliations:** University of Ghana, Ghana

Comment:

Findings presented in this paper are of great public health importance.  Mosi *et al *assessed the microbial content of different brands of sachet water produced, sold and consumed by the majority of the populations in different regions of Ghana with emphasis laid on coliforms and fecal E. coli. They showed that some sachet water brands in Ghana as a whole (since they sampled from 5 geographical distinct regions) are still contaminated by coliforms and fecal E. coli despite awareness-raising from previous reports. However, it would be good to specify the date of water sample collection in the study design to attest that what are referred to as previous reports are really previous reports in term of sample collection period. I will recommend this paper for indexing provided the authors address the comments raised below.

Response:

Comments addressed in manuscript

**Introduction**

Comment:

The authors present sachet water production and consumption at the beginning of the introduction as something specific to Ghana whereas it is common practice in many countries in Africa (West Africa mainly) (Nigeria, Cameroon). It would be good to move from general to specific.

Response:

Comments addressed in manuscript

**Methods**

Comment:

Figure 1 and Table 1 are used in the method section to back up the wider geographic coverage of this study. But there is no link between what is found in table 1 and geographical coverage. The authors should clarify that. Was the map drawn by the authors or it is just an adaptation from another map? If that is the case, then the authors should specify that.

Response:

Comments addressed in manuscript

**Study site and design**

Comment:

Sachet water samples were collected from 41 selected communities. How many samples were collected per community? The reader may have the impression that only one sample was collected per community. If that is the case, why? If many samples were collected then, your sample size should be 41 times the number of sample per brand. The authors should have collected more samples per community and even evaluate the storage conditions in each store, which information would have helped them discuss better their results. Did the authors collect each sachet water brands from the source (packaging company) to rule out the fact that water leaves the company already contaminated? If not, they should put that as a point in the discussion.

Response:

The study was designed to examine the wholesomeness of sachet water in Ghana. Therefore, the target was focused on microbial contaminants at the time it gets to the end user. It would have been interesting to sample water at the source (manufacturing treatment plant), and at the market but it was difficult to incorporate it in the study due to some technical challenges.

Comment:

Giving the brand’s names as Obiri‐Danso *et al* (2003) ^1^ did in their paper “The microbiological quality of drinking water sold on the streets in Kumasi, Ghana” could be of great importance to the consumers in making the choice of which brands to take. Maybe ethically this is not correct but the authors should think about it.

Response:

As rightly said, we considered the ethics of publishing the brand names and decided to leave them out.

Comment:

“These  communities  gave  a  fair  representation of  rural,  urban  as  well  as  peri-urban  communities  in  Ghana  thus giving  the  study  a  wider  geographic  coverage “  the authors mentioned this in their study design, the reader would expect them to present the results and discuss them with respect to level of urbanisation which is not the case in the current version of the paper.

Response:

Comments addressed in manuscript

**Sample collection**

Comment:

-There is a word missing in the following sentence “The most commonly patronized sachet

water  was  determined  after  interviewing  a  number  of  people the  community [...] to central point laboratories within each region where analysis were carried out." There seems to be a grammatical error in this sentence “where analysis was carried out or where analyses were”…

Response:

Comments addressed in manuscript

Comment:

Again, in the methods, the authors did not describe clearly the measures taken to avoid contamination during sample transport and manipulation in the lab which could have contributed to the number of micro-organisms found.

Response:

Comments addressed in manuscript. In addition, transportation, as described by Johnson et al., **was stated in the manuscript which gives reference to the aseptic measures used during the sample collection.

**Results**

Comment:

……E.  coli was estimated  at  1299.7  and  27.5  respectively  with  samples  from the Volta region having more positive total coliforms and E. coli compared  to  other  regions  (Table  1)

There is no link between the sentence and table1. The authors should make sure tables and figures are properly reference in the text.

Response:

Comments addressed in manuscript

Comment:

The title of figure 3 :”Regional analysis of the samples” does not really describe the figure. The reader may think from the title that the figure summarizes the methods used to analyse the sample in different regions and not the analysis outcomes which the authors are referring to.

Response:

Comments addressed in manuscript

**Discussion**

Comment:

The authors could consider adding a small paragraph on the impact of used sachets on the environment, e.g. they constitute breeding sites for mosquitoes such as anopheles which transmit other diseases.

Response:

Since the publication was focused on the microbial contaminants and there was no result or evidence from our study on the environmental impact of sachet water, it would be difficult to discuss the effect of sachet water on the environment and it serving as breeding grounds for mosquitoes. This is however well noted for future studies.

# Peer review: Reviewer response for version 1

**DOI:** 10.21956/aasopenres.13903.r26351

**Published:** 2018-04-25

**Authors:** Olusegun A. Olaoye

**Affiliations:** 1 Department of Food Science and Technology, Michael Okpara University of Agriculture, Umudike, Nigeria

The study presents microbiological analysis of sachet water samples across Ghana, with a view to ascertain their fitness for consumption. The research is an interesting one and of public health significance to the people that consume sachet water in Ghana.

 The study reported occurrence of coliforms and useful microbial indicator of water quality, *Escherichia coli* in some of the water samples analyzed, signaling public health alarm to consumers.

 Authors need to be consistent in their citations/references:, with the use of numbers ( ^1,2^etc) or Surname with years, eg Ngmekpele and Hawkins (2015). Authors should choose a particular style of citation and use such throughout the manuscript.

 Why did authors only take samples for analysis from 5 out of 10 regions? Why were the other regions not considered? Authors should be able to justify this.

 Authors need to discuss briefly the efforts of the water regulatory body in Ghana in ensuring good quality drinking water, especially the regulations to companies involved. Are there laid down regulations by required body, are companies not adhering to such regulations? What possible sanctions can the regulatory body take on erring companies to serve as deterrent to others?

 I suggest that the authors include a Conclusion section.

 I recommend acceptance of the manuscript after the authors address the above points raised and other areas as highlighted in the reviewed PDF article, which can be downloaded here.

I confirm that I have read this submission and believe that I have an appropriate level of expertise to confirm that it is of an acceptable scientific standard, however I have significant reservations, as outlined above.

# Response

**Published:** 2019-01-07

**Authors:** Samuel Mawuli Adadey

**Affiliations:** University of Ghana, Ghana

Comment:

The study presents microbiological analysis of sachet water samples across Ghana, with a view to ascertain their fitness for consumption. The research is an interesting one and of public health significance to the people that consume sachet water in Ghana.

The study reported occurrence of coliforms and useful microbial indicator of water quality, *Escherichia coli* in some of the water samples analyzed, signaling public health alarm to consumers.

Authors need to be consistent in their citations/references:, with the use of numbers ( ^1,2^etc) or Surname with years, eg Ngmekpele and Hawkins (2015). Authors should choose a particular style of citation and use such throughout the manuscript.

Response:

Comments addressed in manuscript

Comment:

Why did authors only take samples for analysis from 5 out of 10 regions? Why were the other regions not considered? Authors should be able to justify this.

Response:

Comments addressed in manuscript

Comment:

Authors need to discuss briefly the efforts of the water regulatory body in Ghana in ensuring good quality drinking water, especially the regulations to companies involved. Are there laid down regulations by required body, are companies not adhering to such regulations? What possible sanctions can the regulatory body take on erring companies to serve as deterrent to others?

Response:

Comments addressed in manuscript

Comment:

I suggest that the authors include a Conclusion section.

Response:

Comments addressed in manuscript

Comment:

I recommend acceptance of the manuscript after the authors address the above points raised and other areas as highlighted in the reviewed PDF article, which can be downloaded here.

Response:

Comments addressed in manuscript
